# Diasporic medical tourism: a scoping review of quantitative and qualitative evidence

**DOI:** 10.1186/s12992-020-00550-x

**Published:** 2020-03-30

**Authors:** Aneta Mathijsen, François Pierre Mathijsen

**Affiliations:** 1 SGH Warsaw School of Economics, Collegium of the World Economy, Aleja Niepodległości 162, 02-554 Warsaw, Poland; 2 ICHEC Brussels Management School, Boulevard Brand Whitlock 4, 1150 Woluwe-Saint-Pierre, Belgium

**Keywords:** Medical tourism, Diasporic medical tourism, Diaspora travel, Diaspora health, Transnational healthcare, Transnational health, Migration health, Medical tourism scoping

## Abstract

**Background:**

There is a growing recognition of the significance of the diasporic dimension of medical travel. Explanations of medical tourism are increasingly presented in a wider context of transnationalism, diaspora and migration. Yet diaspora and cross-border travellers rarely get through the broader narrative of medical travel.

**Objective:**

Our aim in this scoping review was to extend the current knowledge on the emerging subject of diasporic travels for medical purposes. Specifically, we reviewed the existing literature on what is known about the determinants and motivational factors of diasporic medical tourism; its geographic scope and its quantitative estimation.

**Methods:**

Using a scoping review methodology, we conducted the search in seven electronic databases. It resulted in 210 records retrieved. Ultimately, 28 research papers and 6 non-research papers (published between 2002 and 2019) met the following criteria: 1) focus on healthcare and health-related practices, 2) transnational perspective, 3) healthcare consumption in the country of origin (homeland) while being a resident of another country, 4) published in English.

**Results:**

The findings from our review highlighted the importance of diasporic medical patients who had been researched and analysed on four continents. Even though quantitative evidence has been scarce, the data analysed in the scoping review pointed to the existence of non-negligible level of diasporic medical tourism in Northern America, and in Europe. Various motivational factors were enumerated with their frequency of occurrence: medical culture (12), time availability (“by the way of being home”) (9), communication (6), dissatisfaction with the current system (6), healthcare insurance status (5), quality of healthcare (5), second opinion (3), and value for money (3).

**Conclusion:**

Diasporic medical tourists constitute an attractive segment of consumers that is still not well understood and targeted. They are part of transnational communities that cultivate the links between the two nations. They simultaneously participate in bi-lateral healthcare systems via return visits which impact the health systems of sending and receiving countries in a substantial way. In the current globalised, connected and migratory context, transnationalism seems to represent an answer to many local healthcare-related barriers. Sending and receiving countries have put in place an array of programmes and policies addressed to the diasporic medical travellers.

## Introduction

Despite frequent descriptions of medical tourism as the flow of affluent patients from so-called ‘northern’ to ‘southern’ countries (from high-income to low and middle-income countries), there is a growing recognition of the significance of a diasporic dimension of medical tourism (DMT). Explanations of medical tourism are increasingly presented in a wider context of transnationalism, diaspora and migration [1, 2]. Nowadays, migrants remain connected to their countries of origin ‘continuously, instantly, dynamically and closely’ [3] which represents a ‘new form of connectivity’. Their health practices are frequently transnational with healthcare systems and practices being utilised in both countries – of origin and of residence. Given their sheer volume and close attachments to their countries of origin (be it frequent travels, remittances, commercial investments or philanthropy), diasporic medical tourists represent a potentially attractive market for the diaspora’s countries of origin and a challenge to the healthcare systems of the resident countries.

The growing interest in this group of patients is related to the fact that there is a significant increase in the number of migrants across the world. Yet empirical studies related to diaspora and cross-border travellers are scarce, especially in comparison to those treating foreign medical travellers. As Connell [4] puts it succinctly: ‘(…) little has been written on such travellers [diaspora patients returning ‘home’], because they are of limited significance to the industry, not easy to distinguish and even harder to document’.

In general, the relationship between transnationalism and health has rarely been explored in the past [2, 5], and it is just starting to gain attention in the past few years (i.e. [4, 6–8]).

The present article aims at extending the current knowledge on this emerging subject of diasporic travels for medical purposes. We believe that the contribution of this scoping review is twofold: the detailed description of motivational factors for DMT and the quantitative estimation of the size of this market, based on available empirical studies. As for the former: many times in literature motivational factors for DMT are compiled into a few general groups: cultural, linguistic and communication (i.e. [9, 10]). Our scoping review demonstrates that under general headlines, more detailed elements are distinguished. We also attempt to see with which intensity each element appears in the empirical studies. As for the latter: even though analysed research papers applied different methodologies, samples and scopes, the evidence gives certain indication as to the size of DMT. In the discussion section, we will touch upon policies related to diasporic medical travellers from the perspectives of receiving and sending countries.

## Background

### Transnationality and the diasporic dimension of medical tourism

There has been a surge of interest in the subject of diasporas since the late 1980s [11]. It accompanied the phenomenon of the growth in international migration. In 2019, there were 272 million international migrants worldwide (3.5% of the total global population), three times more than in the 1970s [12]. There were around 128 million immigrants (foreign-born people) in the countries belonging to the Organisation for Economic Co-operation and Development (OECD) (over 10% of its population), and 58 million in the European Union (11.5% of its population) [13].

Moving from one country to another involves a change in the relationship towards the country of origin and also the establishment of a new relationship with the receiving country. This process is sometimes referred to as ‘transnationality’ where diasporas constitute potential bridges between two countries [14]. The IOM refers to the diaspora as “individuals and members of networks, associations and communities, who left their country of origin but maintain links with their homeland” ([15]: 28). The project ‘A Europe of Diasporas’ further underlines the aspect of the common identity of groups of people dispersed over several countries [16]. Continuing on this line, we will in the present article use the definition of Constant and Zimmermann [17] who describe diaspora as a “well-defined group of migrants or those with migrant background with a joined cultural identity and ongoing identification (active or dormant) with the country or culture of origin as they perceive it”.

Coles & Timothy [18] conceptualize diaspora as ‘hyphenation’ or ‘hyphenated groups’ (as in the combination of homeland and host state), emphasizing the crucial duality of ethnicity and citizenship. Diasporic movements and regionalized cross-border travels represent bottom-up transnationalism [19]. Travel becomes vital in this process of formation of diasporic networks which are facilitated by return visits [18]. In the world of extensive migration, travel plays a more complex role than just escapism and fun. It enables people to combine leisure with social obligations [20], and with health [21].

The literature suggests that diasporic medical travellers may have constituted a substantial group of medical travellers in certain countries. This phenomenon has been noted in the cases of Colombia, Guatemala, India, Iran, Jordan, Lebanon, Malta, Mexico, the Philippines, and Turkey [21–24]. Puerto Rico, the Philippines, India, Cuba and Taiwan expressly oriented their medical tourism campaigns towards their diaspora populations. As a result, those countries not only benefited from diasporic patients but also from diasporic investment, philanthropy, volunteerism [25], and development via healthcare consumption and production [26].

For the purpose of our article, we will define diasporic medical tourism as follows: “migrants travelling to their country of origin (homeland) and voluntarily using the healthcare there as an act which is planned and/or organized upfront”.

### Diaspora and healthcare services

Given the fact that diaspora members belong to the broad group of migrants, their health needs have been analyzed mostly under the concept of migrant-sensitive health services. Initially driven by research in medical anthropology and immigrant medicine, as well as observations and actions undertaken by front-line healthcare providers and advocacy groups, models of care were designed to meet those health needs. They were mostly adapted to the cultural, linguistic, social, religious, and health status differences that affect the use of mainstream healthcare by diaspora members. The most often-cited health services comprised: provision of interpretation services, language-appropriate written materials, culturally sensitive care - the ability of health care practitioners to acknowledge their own cultural backgrounds, biases, and professional cultural norms - and culturally-tailored health promotion, disease prevention and support programs [27].

Migrants are likely to experience specific challenges impacting their health (in a negative or positive way) due to the conditions surrounding their migration (in the country of origin, in the transit, and in the country of residence). Migration is now acknowledged as one of the determinants of health, among other social factors. There are several theories that attempt to explain this complex relationship between health and migration; among the most important and frequently cited are: the “healthy migrant effect”, theories of ill health, the theory of the negative effect of migration, the allostatic load, and acculturation theories (for a detailed explanation see [28]). However, those theories have some limitations such as the ethnocentric approach using one-sided focus on minorities, difficulties in examining the complexity and diversity of migrant populations as well as the underlying processes [28].

### Diasporic motivations and typologies

Diasporic motivations are seemingly multifactorial. In a comprehensive literature review on migration and tourism, Huang, King & Suntikul [29] cited the following motivational factors for diasporic travel: retaining family ties or fulfilling family obligations (weddings, funerals, and family rituals), visiting friends and relatives, reaffirming social connections and reinforcing their ethnic and cultural identity. For migrants with a longer history of migration, the search for roots and ancestry would have been the primary reason, while for victim diasporas (forced dispersal) it would have been more the spiritual dimension of homecoming, quest or pilgrimage, in which members of diasporas accommodated painful past history or experienced self-transformation (also in [30, 31]).

Little is also known about the typology of the diasporic medical travellers. In the situation where evidence is missing, and working by analogy, we would assume for the sake of the argument that general typologies of the diasporic travellers apply. What is then known about those types of typologies? Various attempts have been made to classify diasporic travellers.

Diasporic travellers are certainly not a homogeneous group. The most frequent and easiest typology relates to the length of the migration experience (those classifiers appear in the research papers analysed in our scoping review). ‘First generation’ (1st) migration refers to foreign-born individuals residing in a foreign country; ‘second generation’ (2nd) to those who are native-born individuals with one or two foreign-born parents; ‘third generation’ (3rd) - those with foreign-born grandparents. Sometimes 1.5th generation may be used to designate foreign-born individuals who migrated to the new country before the age of eighteen and whose behavioural pattern is similar to the second generation. Those groups demonstrate various degrees of attachment to the country of origin. Huang, Hung & Chen’s [30] research indicates that 1st and 4th generation have the highest level of attachment to the country of origin; and the 2nd generation - the lowest. Horsfall’s [32] research indicated that diasporic medical treatment prevailed among the 1st generation, while Nielsen et al. [33], Wallace et al. [34] and Mines, Mullenax, & Saca [35] observed it among 1.5th and 2nd generations.

Diasporic travellers may also manifest various degrees of cultural connectedness to the homeland, as presented in a typology developed by Weaver, Kwek & Wang [36]. Diaspora members who demonstrated a higher level of engagement and deeper cultural connectedness with the country of origin were labelled *the Intrinsics* (high interest in culture and in-depth knowledge, moderate engagement with cultural products) and *the Hybrids* (high interest in culture and high engagement in cultural products). On the contrary, *the Shallows* exhibited little interest and knew little about the home country, and *the Extrinsics* had moderate knowledge about the country. The latter two represented a low cultural connectedness to the country of origin. Thus, it was then suggested that the overseas diaspora may represent a culturally and experientially complex cohort.

## Methods

In this heterogeneous and little researched topic, the method of a scoping review has been of particular interest in collecting and evaluating the current state of knowledge [37]. The aim was to map the literature on this specific research area and identify key concepts, gaps, types and sources of evidence. Specifically, our interest was focused on the following research questions: what is known about the diasporic patients’ experience of medical tourism in terms of: i) conceptualisation, ii) population, iii) dimension, iv) context, and v) determinants.

The search was conducted between November 2018 and January 2019 in the following electronic databases: Emerald Insight, Science Direct, Wiley Online Library, Web of Science, Taylor & Francis Online, PubMed, Google Scholar. An additional search was conducted using the snowballing method. No start date was used. The following keywords and search terms were applied: (‘diasporic medical tourism’ OR ‘diasporic medical travel) AND (‘diaspora healthcare-seeking’) AND (‘immigrant healthcare travel) AND (‘transnational health’ OR ‘transnational healthcare’) AND (‘transnational healthcare-seeking’) AND (‘medical returns’) AND (‘medical homecoming’) AND (‘diaspora patients’ OR diasporic patients’).

The following criteria were applied for including published articles for this scoping review: 1) focus on healthcare and health-related practices, 2) transnational perspective, 3) healthcare consumption in the country of origin (homeland) while being a resident of another country, 4) published in the English language.

**Fig. 1:**
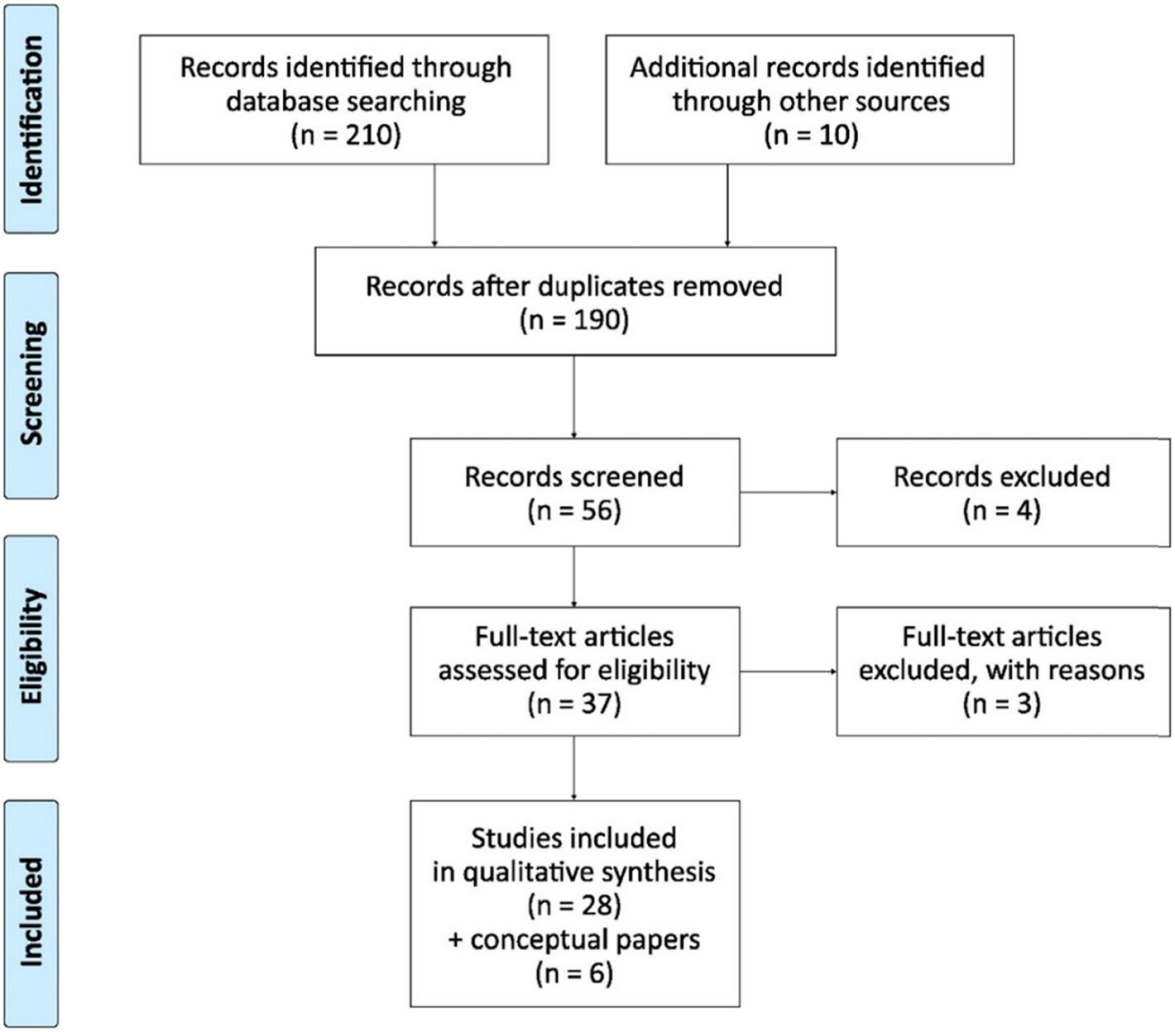
PRISMA flow diagram of the published papers for scoping review. *Source:* Moher, Liberati, Tetzlaff & Altman (The PRISMA Group) [38]

We used a multiple-stage search and review process (Fig. 1). The initial search produced 210 abstracts. After scanning for the above criteria we retained 190 valid abstracts (after removing the duplications). Out of these, a total of 56 articles were included for a full review. We included conceptual notes that we used for further guidance towards research papers. We excluded papers that were focused on healthcare behaviour of immigrants in the country of residence, health worker migration, diaspora health investment, medical tourists, international retirees, refugees and diasporic tourists at large. After a full review, we included in the final selection 28 research papers and 6 non-research papers - literature review, systematic observation and conceptual – that contained data relevant to the research objective.

## Results

### The scope and global distribution of DMT research

The scope of the research spanned peer-reviewed journals related to travel, migration, medicine, healthcare, global health, and sociology. The articles were published in the last two decades, between 2002 and 2019; most of them after 2010.

Researched populations were scattered across the globe. Mexican immigrants were among the most researched populations of all. Table 1 enumerates other populations in detail. Among those, we list: Americans, Brazilian, British, Cape Verdeans, Egyptian, Ghanaian, Indian, Iraqi, Lebanese, Moroccan, Palestinian, Polish, Romanian, Somali, Southern African, South Koreans, Surinamese, Syrian, Turkish, Zambian, and Zimbabwean; with different degrees of specificity. On the other hand, among the countries of residence where the researched diasporic populations lived, we noted: Arab Gulf countries, Belgium, Canada, Denmark, Germany, Ireland, the Netherlands, New Zealand, the UK and the U.S. (Table 1). None of the researched papers analysed populations residing in Asian countries.

**Table 1:**
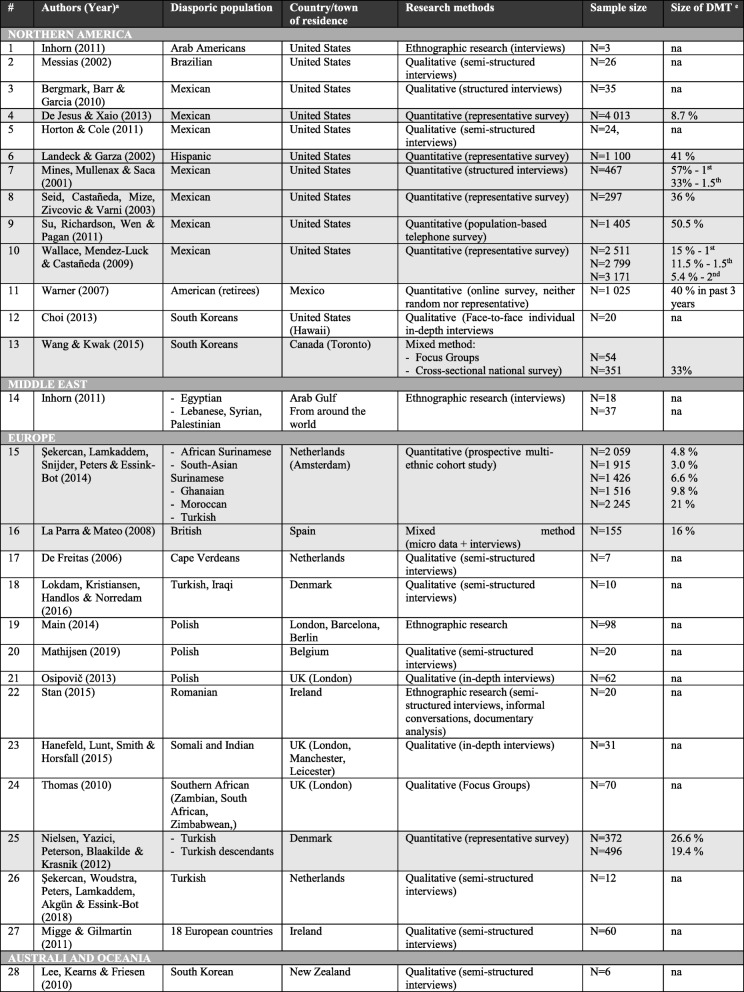
Diasporic populations, their countries of residence, methods (and samples) and authors of the analysed articles (*in grey colour* = quantitative research analysed in the estimation of the DMT volume, available data for past 12 months)

ͣ All reviewed articles are displayed in alphabetical order based on continental regions

ͤ na (non-available)

The qualitative approach was by far the most frequently used method; semi-structured interviews were based on purposeful and convenience sampling, with sample sizes ranging from 6 to 95 participants (*M* = 27). Within the qualitative approach, semi-structured interviews, Focus Groups (FGs), the feminist narrative, and ethnographic methods were applied. Nine research papers were based on the quantitative approach, and data were drawn either from the population-based surveys or collected by the use of questionnaires, with sample sizes ranging from 155 to 4013 respondents (*M* = 1426).

Diasporic medical tourism articles usually dealt with immigrants of the 1st generation. On a few occasions, research was extended to the 1.5th generation [33, 35] and 2nd generation [33, 34, 39–41]. Studies on consecutive generations (3+) were lacking, thus it is impossible to verify whether, for instance, the 3rd generation hypothesis – ‘what the son wishes to forget the grandson wishes to remember’ [42] - is supported or failed.

### Conceptualisation of diasporic medical tourism

Diasporic medical tourists have been framed by many concepts, depending on the domain of the research, be it anthropology, sociology, tourism, geography, economics, health policy, or migration. Scholars differ with respect to conceptual definitions. Various descriptors have been found in the literature to denotate the same group of the researched population: diasporic/diaspora medical tourism or travel [21, 24, 31, 43, 44], diaspora patients [19], patient movements of returners [23], medical homecoming [26], migrants returning for healthcare [45, 46], return reproductive tourism [47], medical returns [2, 39], diasporic medical return [48], use of homeland medical services [45].

The literature related to transnational healthcare treats the subject of diasporic medical travel under the category of transnational healthcare-seeking [2, 49, 50], transnational medical consumerism [51], transnational healthcare practice [50], or healthcare consumption in the country of origin [40]. In fact, the term transnational healthcare-seeking is very often applied to designate analogous behaviour to diasporic medical travel.

Also, there has been no consensus as to the definition. Studies suggested that transnational healthcare was related to simultaneous and potentially frequent participation in multiple healthcare systems what Bell et al. [6] coined as ‘pendular’ and Villa-Torres et al. [8] named as ‘hybridity’ (where migrant goods, ideas and people intermixed between the countries of origin and home). DMT has frequently been defined as a transnational alternative (to the one proposed in the country of residence) to healthcare-seeking behaviour or as a ‘strategy’ undertaken by migrants to obtain healthcare. Therefore diasporic medical tourism was described as a ‘top-up care’ [50], or a ‘safety valve’ [39].

It is interesting to note that the DMT was not the only coping strategy in healthcare-seeking behaviour. The scoping review revealed that the following strategies were implemented by migrants: 1) self-diagnosis and self-treatment as well as self-medication [45, 49], 2) contacting doctors in home countries (via phone, internet, family or friends) [2], 3) delay in seeking primary care [2, 49, 50], 4) practice of emergency-oriented care [49, 50], 5) sharing medication with family and friends [52], 6) alternative, non-biomedical treatment [53].

Given the ontological and definitional discrepancies, the attempts to classify this category of patients have been extremely rare. The scoping review revealed that only two analysed papers presented a typology within which a diasporic category of medical tourists was included. Glinos et al. [23] created a typology of cross-border patients based on two dimensions: the patients’ motivation and the types of funding. Diasporic patients were characterised by the same cultural and linguistic approach to healthcare, i.e. having the same doctor for years, being treated in familiar surroundings, knowing how to make moves in the system (labelled ‘Familiarity’) and presupposition that healthcare was less expensive in the country of origin (labelled ‘No cover’ due to fact of keeping the insurance cover in the home country and /or not being insured in the country of residence). Migge & Gilmartin [46] expanded on the above-mentioned work and highlighted that ‘Familiarity’ might have been misconstrued with miscommunication or lack of appropriate information. However, the cultural and social aspects of healthcare were once more emphasized.

### Determinants (predictors) of diasporic medical tourism

Given that economic reasons drove most of the voluntary migration to countries offering better social and economic conditions, with more advanced healthcare systems, the question arose as to the reasons why immigrants would return to their home countries for healthcare services. As an example, Polish immigrants in Belgium moved between a healthcare system ranked 5th in Europe (Belgian) and a system ranked 32nd in Europe (Polish), where those two healthcare systems differed mostly on accessibility/waiting times, outcomes (i.e. survival rates), range and reach of services provided, and access to certain pharmaceuticals [54].

The literature approached the question from the motivation theory of benefits sought or realised; predominantly the Push and Pull theory defined by Dann [55] and further developed by Crompton [56]. People were pushed or pulled by the forces of motivations and destination attributes to effectuate travels. Push factors were often socio-psychological constructs of the subjects and their environment, while Pull factors were the attributes of the destination that made it attractive to travel and choose a specific destination.

The following Push and Pull factors were enumerated as a result of the scoping review. They have been presented in order of frequency of occurrence (a summary of relevant studies in Table 2):

**Table 2:** Determinants of DMT according to their number of citations in the analyzed articles

FACTOR (frequency of occurrence) *	RELATED ARTICLES
Medical culture (12)	Bergmark et al., [57] ; De Freitas, [58] ; Horton & Cole, [39] ; Inhorn [47] ; Lee et al., [45]; Mathijsen [44] ; Messias [52]; Mines et al., [35] ; Osipovič, [50] ; Seid et al., [59] ; Şekercan et al., [40] ; Wallace et al., [34]
Time availability (‘by the way of being home’) (9)	Bergmark et al., [57]; Hanefeld et al, [60] ; Lee et al., [45]; Main, [61]; Mathijsen [44]; Messias, [52]; Osipovič, [50]; Seid et al., [59]; Şekercan et al., [62]
Communication (6)	De Jesus & Xiao, [63] ; Lee et al., [45] ; Main, [61]; Mathijsen [44]; Su et al., [41] ; Wang & Kwak, [2]
Dissatisfaction with current system (6)	De Freitas, [58]; Main, [61] ; Migge & Gilmartin, [46]; Nielsen et al., [33]; Osipovič, [50]; Şekercan et al., [40]
Healthcare insurance status (accessibility) (5)	Choi, [49]; Seid et al., [59]; Su et al., [41] ; Wallace et al., [34] ; De Jesus & Xiao, [63]
Quality of healthcare (5)	Bergmark et al., [57]; De Jesus & Xiao, [63] ; Lokdam et al., [64]; Su et al., [41]; Wang & Kwak, [2]
Second opinion (3)	Lokdam et al., [64] ; Mathijsen [44]; Şekercan et al., [40]
Value for money (affordability) (3)	Mathijsen [44]; Migge & Gilmartin, [46] ; Stan, [65]

*When there is an equal number of references to a specific factor, alphabetic order is applied

#### Medical culture

‘Medical culture’ was construed as accordance between the cultures of both patients and providers [34]. It encompassed a way of thinking and a very country-specific medical culture, personal attention to the patient, and familiarity with the system. On a negative note, when there was not this level of understanding, the immigrants clearly spoke about feelings of being a *misunderstood outsider*, or *an immigrant on the margin* [52]. As a result, the perceived cultural distance from the healthcare system in the country of residence was significantly associated with the utilisation of healthcare in the country of origin [40].

Bergmark, Barr & Garcia [57], Horton & Cole [39] and Seid, Castañeda, Mize, Zivkovic & Varni [59] demonstrated a clear preference for the healthcare of the country of origin among Mexicans residing in the U.S. Immigrant farmworkers who spent the majority of their adult lives in the United States purposely sought a ‘Mexican Medicine’ that was understood as rapid diagnosis with few laboratory tests, of common linguistic and cultural background, where no patients’ records were kept, and the receipt of medicines was prompt and had immediate effects [35]. In other instances it was described as patient-centred care which meant more time dedicated for speaking with and examining the patient, direct physical touch, rapidity of services. The U.S. care (country of residence) was criticized for frequent referrals and tests, impersonal doctor-patient relationships, uniform treatment protocols and reliance on surgery, as well as difficulties in seeing a doctor at the weekend [39]. The immigrants also complained about over-reliance of the physicians on certain medication which they perceived as symptomatic of inadequate care [39, 50, 52, 58].

The medical culture was even more important in the field of highly specialised medical interventions, such as reproductive medicine. Inhorn [47] studied Middle-Eastern diaspora members who were looking for reproductive healthcare services. The importance of the country of origin was justified by the search of the donor with the same origins as theirs, without any risk of making a mistake that the child would look culturally different. They chose the comforts of home country also for the reason of avoiding any kind of discrimination and cultural insensitivity. They also believed in what the author labelled as ‘medical-expatriotism’ - the feeling of relative superiority of home-country medical services versus those in the resident country (without further elaboration in how that superiority manifested itself).

The ability to understand the medical terminology, instructions and explanations (labelled ‘the language of medicine’) and the importance of familiar surroundings and home environment (named ‘comforts of home’) were other motivational factors for this kind of DMT [47]. The latter two were supported by the research conducted on Polish diaspora in Belgium in a more generalized medical context [44].

‘Affective’ healthcare was equally important as ‘effective’ healthcare [45]. The experiences of trust and familiarity were significant factors influencing the well-being of immigrant patients [44, 45, 50].

#### Time availability (“by the way of being home”)

In many instances, diasporic travellers used healthcare services back in their countries of origin while conducting the travel labelled in the scientific literature as ‘Visiting Friends and Relatives’ (VFR) [44, 45, 57, 61, 62]. This factor of time availability was termed by certain authors as *by-the-by* factor [44], or *aprovechar* (meaning ‘to take advantage of’) in reference to the Mexican context [57] or *add-on* treatments [60]. Diaspora members were regular visitors to the country of origin who on the trip happen to ‘add on’ medical services. They tended to sketch a ‘To do list’ where among few regular things to do appeared medical services [44].

In general, what facilitated diasporic travel (or mediated it) was social capital - social connections or social networks back in the home country [66] - and the spatial capital - the possibility of travelling low-cost and to relative geographic proximity [50]. In case of the diasporic travellers, their principal motivation to travel to the country of origin was visiting family and friends. And what allowed them to do so, was a geographical proximity perceived as travel convenience and travel time.

#### Communication

Tied to the factor of the Medical culture, language proficiency and communication skills appeared to be important Push factors (− .13, *p* < .05 in [63]; also in [61]). The relationship between fluency in language and the use of healthcare was established in Su et al., [41]. Wang & Kwak [2] reported that, when language became a barrier for Korean immigrants in Toronto (Canada), the help of translators was fundamental. Difficulty in describing symptoms, as well as understanding doctors’ instructions and highly specialised medical language were expressed by long-term and recent migrants. As a result, almost all the participants preferred to have a Korean-speaking family physician, and 6 out of 10 were able to have one. This was also supported by the findings of Lee et al. [45] for whom the linguistic style (less authoritative and more probabilistic) and the speech mannerism (the way the diagnosis was delivered) by the General Practitioners (GPs) in New Zealand created within Korean immigrants an important cultural barrier leading to possible anxieties. Mathijsen [44] pointed out that the capacity to understand medical terminology, instructions and explanations was even more important than foreign language proficiency.

Given the importance of obtaining the right diagnosis and understanding well the proposed treatment, diasporic medical travellers opted for places and solutions that ensured proper communication. Healthcare domain revealed itself to be a linguistically-sensitive field.

#### Dissatisfaction with the current system

The main Push factor consisted of resentment towards the local healthcare system. Danish studies demonstrated that what led foreign-born patients to use foreign healthcare was dissatisfaction with a local system coupled with longer stays in their home countries [33]. The lack of information on the available healthcare services represented a barrier for Cape Verdeans accessing healthcare in the Netherlands [58].

In many instances, healthcare providers in the country of residence were perceived as untrustworthy, disinterested and negligent [46, 61]. Cape Verdeans patients in the Netherlands experienced feelings of rejection, vulnerability and neglect [58]. In the same manner, Polish immigrants in the UK experienced a sense of unease and a lack of amenability in contacts with the local health system (NHS) [50]. Thus, they perceived doctors in the country of origin to be more trustworthy.

GP gatekeeping and lack of direct access to a specialist in the country of residence discouraged Polish [61] and Turkish [40] immigrants. Turkish immigrants were more likely to visit private healthcare clinics in Turkey, where the local context enabled them to overcome GP gatekeeping and opened the possibility of seeing the specialist in a fast and efficient manner.

#### Healthcare insurance status (accessibility)

In Seid et al. [59], Su et al. [41] and Wallace et al. [34] health insurance status was the primary reason for using Mexican physicians by diasporic medical travellers. De Jesus & Xiao [63] supported the hypothesis that the lack of continuous health insurance coverage was one of the stronger predictors of healthcare-seeking in Mexico or any other Latin American country (− .15, *p* < .05). DMT enabled U.S. Hispanic residents to overcome healthcare accessibility barriers in the U.S. The lack of local insurance also seemed to be a reason for diasporic medical tourism in relation to Korean immigrants in Hawaii [49]. Yet, it was not always the case for immigrant patients - Polish diaspora in Belgium continued to use the healthcare services in the country of origin despite their insurance in the country of residence [44].

#### Quality of healthcare

The quality of available healthcare represented yet another motivational factor for DMT (.13, *p* < .05 in [63]). A perceived lack of quality healthcare led to dissatisfaction with healthcare in the country of residence [41, 64].

The quality level of healthcare was judged on the swiftness of obtaining healthcare services, answers, and treatments. Mexicans in the U.S. and South Koreans living in New Zealand and the United States were motivated by receiving faster service (getting the service even on the day of arrival) and having access to hospitals equipped with the latest medical technology [45, 57].

Certain medical interventions were deemed to be of a higher standard and more affordable back in the home countries [2]. For Koreans living in Toronto, the quality of service was judged by the possibility of obtaining a comprehensive medical examination back in the country of origin (including MRI, ultrasound or mammography) which was not covered by the publicly-funded system in the country of residence (Canada).

Notwithstanding, for Migge & Gilmartin [46] the quality issue was double-edged. According to their research, the immigrants in Ireland rather seemed to be confused about the Irish healthcare system, their entitlements and available services – a reason for which they misjudged the quality.

#### Second opinion

Lokdam et al. [64] and Mathijsen [44] substantiated that one of the reasons for DMT was a search for a second opinion. This factor was also highlighted among descendants of Turkish immigrants in Netherlands who seemed to be familiar with the Turkish healthcare system due to their regular VFR travels. They knew how to navigate both systems (Dutch and Turkish) and they chose the best and fastest treatments [40]. Transnationalism often enabled migrants to seek a second opinion or confirmation of the diagnosis in very different healthcare environments and systems.

#### Value for money (affordability)

In the literature related to foreign-patients medical tourism, the cost factor seems to be the most frequently cited motivation factor. Yet, for the diaspora population, it did not seem to be the case. Only in particular situations, where the savings seemed to be very substantial, it did represent a driving factor, otherwise it was usually coupled with time availability (“by the way of being home”) and cultural affinity factors.

The affordability Push factor was described by DMT either as a concept of value-for-money [46] or a relative cost mitigated by the costs of transport and accommodation [44]. Immigrants in Ireland judged the costs of healthcare services in relation to their level of quality and availability as inadequate. Romanian residents in Ireland boosted the choice they had to return to their home country for healthcare services; they had a choice because they could afford it [65]. Poles living in Belgium most often quoted ‘relative cost’ in conjunction with dentistry and orthodontic healthcare where savings seem to be substantial [44].

### Quantitative estimation of diasporic medical tourists

The papers analysed in this scoping review gave a certain indication as to the estimated volume of DMT, a question that has often been raised. We looked at the data from the sound quantitative research (Table 1 highlighted in grey), however these numbers should be interpreted with caution given the limited number of studies and their different methodologies and samples.

**Fig. 2:**
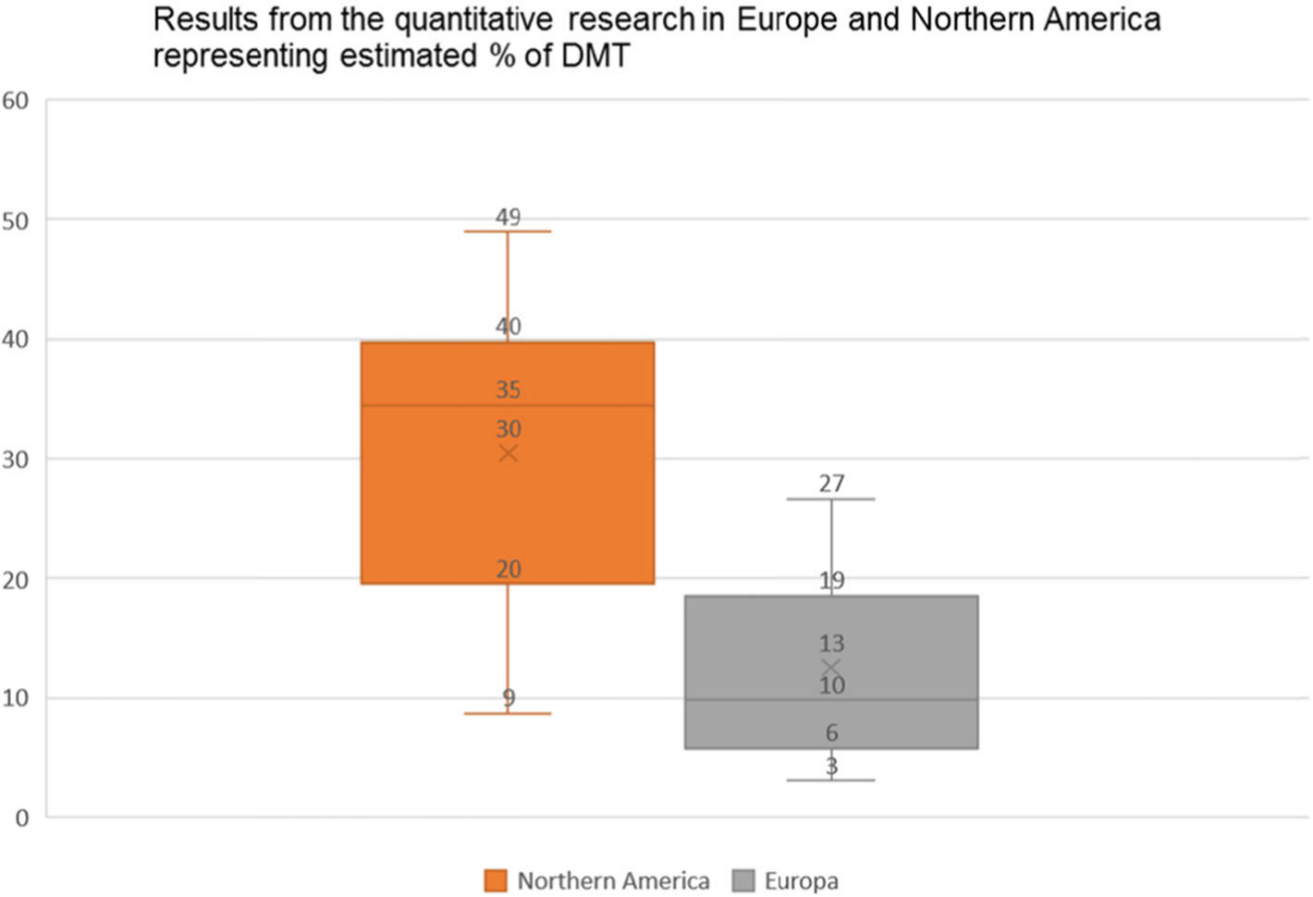
Boxplot representing the estimated % of the DMT – in Europe and in the Northern American continent - from the quantitative studies revised under this scoping review

The boxplot (Fig. 2) representing the results of the quantitative research conducted in the Northern American continent (mostly among Mexicans residing in the USA), was comparatively tall, which suggested that the estimated per cent of diasporic medical tourists varied quite extensively (from 8.7 to 57%) yet the median was relatively high (35%).

Firstly, in the less-recent articles the numbers were much more substantial. The research of Landeck and Garza [67] indicated that about 41% of the Laredo U.S. Hispanic residents were using cross-border physician healthcare services in Mexico. In Seid et al. [59] 36% of the children of Latino farmworkers received their healthcare in Mexico. In the case of the Binational Health Survey 50% of the respondents indicated a preference for Mexican healthcare; and when treatment was sought, 57% actually used healthcare in Mexico [35]. This preference tended to fade away with time, but even among those who had spent more than three-quarters of their adult lives in the United States, one-third would still have opted for healthcare in Mexico [35]. Warner & Jahnke [68] estimated that $100–300 million per year was spent by Mexicans living in the US and returning to Mexico for healthcare.

However, in more recent research the quantitative estimations were lower, with one exception for Su et al. [41] where 50.5% (*N* = 1405) used healthcare services back home in the past 12 months. In the study of Wallace et al. [34] - 15% of Mexican immigrants were using medical healthcare back in Mexico (*N* = 8481), and that of De Jesus & Xiao [63] - only 8.7% of citizens of Mexican-origin used healthcare in their country of origin (*N* = 4013). Nevertheless, when the latter percentage was translated into numbers, it represented 4.5 million Hispanic adults.

Conversely, among American retirees living in Mexico, 17% spent at least one day in a U.S. hospital and 40% had visited doctors in the U.S. in the last 3 years, which amounted to $500–$1000 per person [69].

As for the South Korean immigrants from Canada (Toronto) who had visited Korea in the past 12 months, one-third (33%) mentioned using health services in Korea, either for themselves or by their family members [2].

The research conducted on immigrants in Europe also gave a hint as to the quantitative dimension of DMT, yet a number of quantitative studies with substantial population sample has been very scarce.

The boxplot (Fig. 2) representing the results of the quantitative research conducted in Europe was comparatively short, which suggested that the estimated per cent of DMT didn’t vary extensively (from 3 to 27%), yet the sections were uneven in size, with median significantly low (10%) suggesting that there were two groups of results: one below 10% (African and Asian diaspora in Europe) and another one above 16% (Turkish and British diaspora in Europe).

La Parra and Mateo [70] looked into British nationals residing in Spain and found that 27% were eligible for National Health Service (NHS) care but, in reality, only 16% of the British residents on the Costa Brava visited a GP in the UK, and an additional 9% had appointments in both countries (however, more detailed information regarding other interventions is missing). The available data showed that also the Turkish population demonstrated an important activity of searching for healthcare services back in the country of origin: 21% of Turkish immigrants in the Netherlands (Amsterdam) declared healthcare consumption in the country of origin in the past 12 months [40]. Additionally, the study conducted in Denmark demonstrated that 27% of citizens of Turkish origin used healthcare in a foreign country [33]. Even though the above-mentioned research investigated healthcare use in ‘a foreign country’, not necessarily in the country of origin, both were akin. Moroccans and Ghanaians residing in the Netherlands displayed certain DMT activity (past 12 months), at 10 and 7% respectively [40]. As suggested by the authors, the distance to the country of origin and the travel cost might have been significant factors of this size of DMT. Surinamese from Africa and South Asia demonstrated very low DMT activity: 5 and 3% respectively.

Moreover, not conclusively but indicatively, the narrative from the qualitative studies pointed to the importance of the DMT. Osipovič [50], researching the usage of healthcare services by Polish immigrants in London, clearly stated that accessing those services in Poland was done by the majority of research participants; and on a regular basis. In line with this research, Mathijsen [44] noted that even though its population sample did not allow generalization, three-quarters of the respondents in the research used healthcare services in their country of origin.

## Discussion

In this review, we looked into a handful of empirical studies and a few important conceptual articles that explicitly treated the interplay of health, migration and tourism. Importantly, few of these studies were quantitative, or on significant population samples (the largest being over 4000).

The findings highlight the importance of the segment of diasporic medical patients. They have been researched and analysed on four continents (resident countries in Europe, Middle East, Northern America and Australia/Oceania), yet data for Asia was missing. This concurs with the growing interest in diasporas and transnationalism over the past ten years.

Diasporic medical tourists were descried as pendular due to their transnational and simultaneous way of participating in multiple healthcare systems utilised often as a strategy to obtain desired healthcare. Multiple motivational factors for this behaviour were identified with their frequency of appearance on the scoping review. This scoping review demonstrated that the consumption of healthcare represented one of the explicit reasons for diasporic travel (VFR) and that various motivational Push and Pull factors (theoretical foundations explained earlier) existed.

Among Pull factors, the researchers pointed to the cultural dimension of healthcare, be it medical culture, communication ability or the affective aspect of healthcare. Moreover, the role of comparative knowledge was underlined; diasporic medical tourists were motivated by the possibility of obtaining a second opinion or alternative care. They also paid attention to the relative costs, weighing all available options. Lastly, time availability and sunk costs (travel for VFR reasons) motivated them to use the healthcare of the country of origin.

The lack of health insurance/coverage and the absence of specific care in the country of residence were highlighted within Push factors. Additionally, negative experiences related to the cost or quality of the healthcare system in the country of residence and perceived cultural distance represented barriers to healthcare utilisation.

Foregoing data about the size of the market have suggested that the scale of DMT may be important in volume. The previously mentioned studies indicated that in the Northern American continent - i) between 8.7 and 57% (median 36%) of Mexican diaspora population, and ii) 33% of South Korea diaspora in Canada - used healthcare services back in their home country. In Europe, the percentage oscillated between 3 and 27% (median 10%), with the population of Turks (residing in Denmark or the Netherlands) and Brits (residing in Spain) demonstrating higher tendency to undertake DMT than the population of the Moroccans, Ghanaians, and Surinamese.

### Policy and managerial implications

Nation states have been pushed into moving to a new form of global citizenship [71] extending their boundaries to incorporate politically and socially diaspora members. This transnationalism - explained earlier as potential bridge building between diasporas and countries of residence - has been extensively researched in sociology and anthropology over many years; yet in public health, this subject has been mainly researched only since 2010 [8].

Ormond & Lunt [9] suggest that countries with robust welfare systems are directing their attention as to how to respond to the situation of transnationality of their citizens, while countries with declining welfare systems are trying to benefit from it. The model of ‘diaspora as resource’ translates into an increased engagement by the states with their populations abroad [72]. In our very specific case of the diasporic medial tourism, it has been noted that certain countries included diaspora members into the national medical tourism strategies i.e. the Philippines, South Korea, Trinidad and Tobago; they targeted their medical tourism campaigns to the diaspora [73, 74]. As a title of example, DMT has been promoted and advertised to the diaspora populations via ethnic networks and community media e.g. to the Filipinos living in the U.S. [75] or to the South Koreans living in the United States and Canada [2, 74]. Moreover, medical services have been advertised to diaspora members by third countries (e.g. German clinics that advertised on Somali television channels or Gujarat clinics to Non-resident Indians) [76]. Also, local community networks played a role in healthcare information dissemination [53].

Diasporic medical tourists - perceived as a resource by home countries - are appreciated as ‘early adopters’; testing the services and passing on their experiences to people around them in resident countries [24, 26]. They also continue to go back to their countries of origin even when the situation in the country is not very stable [24].

The private sector is also interested in benefiting from diaspora as a resource. There have been first attempts to develop diaspora-specific insurance schemes, i.e. bi-national health insurance plans developed in California to cover Mexicans working in the U.S. and using Mexican medical facilities (i.e. ‘Salud con Health Net’ developed in 2000) [77].

Concomitantly, in the resident countries, states grapple with adequate healthcare service delivery to the migrants (so called migrant-sensitive healthcare). That is why diaspora organizations often act as supportive partners in defining migrant needs and offering expertise and healthcare services e.g. the Uganda Diaspora Health Foundation developing culturally appropriate mental health care in the UK [78]. They act as catalysts for their community members. Migrant-sensitive health systems and programmes are being introduced in the countries of residence with the aim of integrating those needs into healthcare planning, financing, policy, implementation and evaluation, i.e. ‘Migrant Friendly Health Centres’ in Spain or ‘Migrant Friendly Hospitals’ in Europe [79]. Phillimore et al. [7] provides an extensive overview of responses and policies addressing migrant and minorities healthcare issues, reframing it as rather ‘patchy’- temporary, and rather reinforcing a model of migrant or minority pathology.

### Limitations

This review has limitations. Firstly, only English-language sources were retrieved and reviewed. This could also be a potential explanation as to the scarcity of Asian related studies. The second limitation could be a methodological nationalism - the fact that numerous studies are related to the Mexican population residing in the United States. The weight of evidence may be skewed towards a specific population but papers across the globe demonstrated corresponding motivational factors. Thirdly, as articles were published solely in the last two decades, between 2002 and 2019; most of them after 2010, even though the interest in diasporas dates back to the 1980s. Lastly, the scoping review does not analyse the nature of the treatment of DMT due to the inconsistency of classification and categorization of healthcare services (multiple approaches have been applied and in many cases the detailed explanation is missing). One over-arching conclusion that we could probably draw from the analysed data is that diasporic medical tourists travel for non-emergency, straightforward procedures, in line with Connell [19].

### Future research directions

Future research may focus on further quantifying of diasporic medical tourism, especially in the region of Asia where data is lacking, or not available in English. Moreover, an estimate of the value of diasporic medical tourism would be of great use. For these reasons, a better understanding of its frequency and of the nature of the use of diasporic healthcare services is needed. Also, comparative research on the DMT of different migrant groups should explore distinct characteristics and motivational factors. Longitudinal studies would greatly contribute to the matter – whether diasporic medical tourism changes over time, when the shift of enculturation occurs (ceasing diasporic travels), and how it applies to various generations of diasporas.

Conceptual and theoretical discussions are generally rare. Most of the research is conducted from a practical perspective and from the point of view of the receiving country. Little research exists from the perspective of the home country. Arguably, this field is still at the early stages of research without a coherent system of knowledge. It rather presents a patchwork of conceptualizations coming from three research domains: health, migration and tourism.

There is also a need, particularly for the marketing campaigns already applied by home countries, to evaluate communication campaigns targeted on this sub-segment: how successful diasporic offers have been so far, how well communicated, and how effective. Further elaboration on the process and determinants would benefit those targeting this segment of the market.

## Conclusion

Our scoping review mapped the evidence related to the diasporic medical tourism which will most likely continue to grow given increased migration and global developments [31]. As migration and travel continue to grow and return visits of so-called ‘hybrid’ citizens [8] continue to increase, future research can develop and flourish. In the current globalised, connected and migratory context, transnationalism seems to be the answer to many local barriers.

Diaspora travellers constitute an attractive segment of consumers that is still not well understood and targeted. They are part of transnational communities that cultivate the links between two nations. They simultaneously participate in multiple healthcare systems via return visits effectuated for the purpose of maintaining family and social ties, reinforcing their ethnic and cultural identity, and reaffirming connections.

Eight motivation factors for DMT were identified (in the order of frequency of occurrence): medical culture, time availability (“by the way of being home”), communication, dissatisfaction with the current system, healthcare insurance status (accessibility), quality of healthcare, second opinion and value for money (affordability).

More importantly, even if quantitative research is scarce, the data analysed here suggest that diasporic medical tourism may be non-negligible in numbers, which has been demonstrated with the indicative quantitative studies across the Northern American continent and Europe.

## Data Availability

Not applicable.

## Abbreviations

DTM: Diasporic medical tourism
GP: General practitioner
VFR: Visiting friends and relatives
